# Communicable Disease Surveillance in Lebanon during the Syrian Humanitarian Crisis, 2013–2019

**DOI:** 10.3390/epidemiologia4030026

**Published:** 2023-07-03

**Authors:** Zeina Farah, Majd Saleh, Hala Abou El Naja, Lina Chaito, Nada Ghosn

**Affiliations:** 1 Epidemiological Surveillance Program, Ministry of Public Health, Beirut 2832, Lebanon; 2 London School of Hygiene and Tropical Medicine, Department of Global Health and Development, 15–17 Tavistock Place, London WC1H 9SH, UK

**Keywords:** Lebanon, communicable diseases, public health surveillance, disease outbreaks, refugees

## Abstract

Lebanon has been one of the most affected countries by the Syrian humanitarian crisis. The national communicable disease surveillance was enhanced to detect outbreaks among Syrians. In this study, we aim to describe the findings of the communicable disease surveillance among Syrians in Lebanon, compare it to residents’ data, and describe the implemented surveillance activities between 2013 and 2019. During the study period, data on communicable diseases was mainly collected through the routine national surveillance system and an enhanced syndromic surveillance system. Predefined case definitions and standard operating procedures were in place. Data collection included both case-based and disease-specific reporting forms. Descriptive data and incidence rates were generated. Information was disseminated through weekly reports. Activities were conducted in close collaboration with different partners. The most commonly reported diseases were: viral hepatitis A, cutaneous leishmaniasis, mumps, and measles. Hepatitis A incidence increased in 2013 and 2014 among Syrians as well as residents. For leishmaniasis, the incidence increased only among Syrians in 2013 and decreased after that. An outbreak of mumps was reported among Syrians between 2014 and 2016, with a peak in 2015 concomitant with a national outbreak. Outbreaks of measles were reported among Syrians and residents in 2013, 2018, and 2019. The infrastructure of the well-implemented surveillance system in Lebanon has been utilized to monitor the health status of Syrians in Lebanon, early detect communicable diseases among this population, and guide needed preventive and control measures. This highlights the importance of having a flexible surveillance system that can be adapted to emergencies and the importance of sharing results with involved partners.

## 1. Introduction

Lebanon is one of the most affected countries by the Syrian displacement due to the large number of refugees post Syrian war in 2011 [1]. The number of registered refugees reached more than 1 million in April 2015, making Lebanon the country with the highest number of refugees per capita [2]. The Syrian refugees have taken shelter mostly within governorates bordering Syria, for instance, 39% resided in the Great Bekaa province (includes Bekaa and Baalbak/Hermel) and 27% in the Great North province (includes North and Akkar) [1]. Based on a vulnerability assessment of Syrian refugees in Lebanon in 2018, over half of the refugees were children, fifty four percent of them were below 18 years of age, 44% between 18 and 59 and around 3% were above 60 [3]. Most refugees were integrated into the local community: 73% in residential buildings, 17% in ad-hoc informal settlements and 9% in non-residential buildings [4]. Further, the average household size for Syrian refugees has changed over the years: 7.7 individuals in 2013, 5.3 in 2015, and reaching 4.9 members in 2017 and 2018 [3].

According to the University College London (UCL)–Lancet Commission on Migration and Health, infectious diseases pose a high burden on migrants in several settings [5]. Preventive and curative services are needed to ensure early detection and treatment of infections in migrants. Thus, data on migrant health is needed to design targeted interventions in order to contain outbreaks and prevent new infections [5]. Following refugee inflow, epidemiological surveillance for data generation and response measures in host countries must adapt to new challenges and face new threats. To plan appropriate surveillance activities for refugees, it is crucial first to have good understanding of the most common communicable diseases in refugee settings and good knowledge of the surveillance methods to be implemented in such settings, yet, published data on how these surveillance activities for refugees take place is minimal worldwide [6]. Publishing data on surveillance activities and their findings is crucial to fill the gap in the literature and help countries facing similar situations to set up or enhance their surveillance systems.

In Lebanon, as fifty three percent of Syrians reside in inadequate shelter conditions [4] increasing the risk of communicable disease transmission, rapid detection, and prompt response to epidemics among this displaced population is crucial. The early warning system for communicable diseases in Lebanon is integrated within the national communicable surveillance system operated by the Epidemiological Surveillance Unit (ESU) at the Ministry of Public Health (MOPH). Following the Syrian humanitarian crisis, various activities have been implemented to enhance and strengthen the surveillance and response capacity especially that, refugees are integrated in the local community and seek medical services mainly at existing health facilities. Below we aim to describe the main surveillance findings for the Syrian population in Lebanon between 2013 and 2019, i.e., pre-COVID-19, compare it to the resident data and describe the implemented surveillance activities. 

## 2. Materials and Methods

### 2.1. Indicator-Based Surveillance

Data on communicable diseases among all residents in Lebanon including Syrians was mainly collected through the indicator-based surveillance (IBS) which includes the routine national surveillance system and the syndromic surveillance system.

#### 2.1.1. Routine Surveillance System

Case definitions and standard operating procedures (SOP) for surveillance and investigation guided collection of routine surveillance data. This required filling the following forms: (1) a case-based form targeting forty diseases or syndromes reported immediately or weekly; or (2) disease specific reporting forms for poliomyelitis/acute flaccid paralysis, measles and meningitis (all reporting forms are posted on the MOPH website https://www.moph.gov.lb/en/Pages/2/193/esu (accessed on 25 April 2023)). The list of mandatory notifiable diseases is regularly updated by MOPH upon national, regional, and international needs. Data sources were all healthcare facilities in both public and private sectors including hospitals, medical centers, and laboratories. 

Reporting forms were filled by healthcare providers and sent by fax to the MOPH district level. At this level, forms were verified, and investigation was initiated using disease-specific forms. Reporting and investigation forms were transmitted to the provincial and central levels. At the central level, data was managed using Epidata 3.1 and EpiInfo 6softwares, feeding into the national database system which includes all surveillance data collected from the different data sources. Descriptive analysis by time, place, and person was performed and incidence rates were calculated. Population estimates were generated using available figures from the Central Administration for Statistics, the statistical department at the Ministry of Public Health, the United Nations Relief and Works Agency (UNRWA) for Palestinian population in Lebanon, and the United Nations High Commissioner for Refugees (UNHCR) for the Syrian population in Lebanon. Alerts were generated using fixed thresholds for rare diseases and the historical 5-year averages for endemic diseases. 

During the study period, ESU was conducting several activities aiming to enhance and monitor the reporting process. For instance, all health facilities in Lebanon from both public and private sectors had a designated focal person in charge of reporting communicable diseases and coordinating with the ESU team. The focal person is responsible for searching for communicable diseases in the facility and report to ESU on weekly basis the presence or absence of communicable diseases, through the weekly hospital zero-reporting system. On the other hand, the active surveillance system which entails regular field visits of ESU personnel to selected hospitals on weekly basis, was used to check the hospital admission registry for any missed communicable disease case and sensitize hospital staff members. Regular follow up and coordination with heath facility focal persons was also maintained. 

ESU notification system covered all Lebanese areas and all residents regardless of their nationality. However, with the Syrian humanitarian crisis, some reporting and investigation forms were amended to enhance the quality of collected data among the refugee population. For instance, the nationality of the patient was added to the reporting forms and a specific reporting form was developed for measles. Moreover, the detailed locality of the patient and the code of the informal settlement was added to the hepatitis A investigation form to facilitate the detection of alerts and referral to the Water, Sanitation and Hygiene (WASH) sector. 

#### 2.1.2. Disease-Specific Surveillance Systems within ESU

The acute flaccid paralysis (AFP) surveillance system was also enhanced with the beginning of the Syrian humanitarian crisis especially after the detection of poliomyelitis cases in Syria. In 2014, new staff was recruited to support the enhancement of the system. The used case definitions were adopted from WHO guidelines. AFP case investigation included data collection, specimen collection, laboratory testing, follow up and case classification. For “hot cases” or cases with incomplete vaccination, suspicion of polio by physicians, travel history to polio countries (including Syria in 2013 and 2014) or cluster of cases, a rapid vaccination coverage survey was conducted around the area of the patient. Investigation findings were documented using specific forms (investigation form, specimen form, rapid coverage survey form, follow up, classification). 

Moreover, in 2016, with the support of WHO, the environmental surveillance system for poliomyelitis was initiated aiming to detect the presence of any wild poliovirus or vaccine derived poliovirus (VDPV) and ensure the absence of polio circulation in Lebanon. Through this system, specimens were collected from sewage plants on a monthly basis. Provinces highly populated with Syrian refugees were selected. One specimen was collected per site per month. During the study period, 4 sites were active covering the Great Bekaa, the Great North and Mount Lebanon provinces. 

Similarly, the measles surveillance system was enhanced. In 2013, a specific reporting form for measles was developed including variables on vaccination status, clinical manifestation and specimen collection in addition to demographic data. This aimed to improve the quality and the completeness of data collected from patients by focal persons especially that patients in remote areas and refugee camps might be unreachable through a phone investigation. Upon detection, specimens were collected from suspected cases. This includes serum and oral fluid specimens. The later were mainly used for out-patients and cases in refugee settlements. 

#### 2.1.3. Syndromic Surveillance

After the refugee crisis, the syndromic surveillance system, initiated in 2006 aiming to collect data from primary health centers, was enhanced. New reporting sites were added such as the medical mobile units (MMU) which are run by national and international non-governmental organizations (NGOs) to provide medical services mainly to Syrians living in informal tented settlements and remote areas. Through this system, data was collected using an aggregate-data reporting form including a list of syndromes for two age groups (<5 years and ≥5 years). Further, after the refugee influx, leishmaniasis was added to the reporting form. In 2017, reporting was computerized using DHIS2 software to ensure timely reporting and data accessibility. Data collected through this system was analyzed, alerts were generated, and weekly bulletins were prepared.

### 2.2. Sensitization Activities

In order to improve the reporting of communicable diseases among refugees and the host community in Lebanon, regular training sessions were provided to healthcare facility staff and surveillance teams at the central and peripheral levels. In addition, since 2015, training sessions on communicable disease reporting were conducted for UNHCR refugee outreach volunteers (ROVs) serving the Syrian displaced population in order to sensitize them on specific syndromic case definitions, highlighting the risk of communicable diseases in informal tented settlements and the importance of reporting cases of AFP, measles, mumps and cholera in these settings. 

### 2.3. Data Sharing and Collaboration

As part of the enhancement of the early warning system in Lebanon following the Syrian refugee crisis, surveillance guidelines with case definitions and reporting forms were developed. These guidelines were distributed to all reporting sites and posted on the MOPH website. Moreover, a specific section on surveillance data among refugees was added to the MOPH website. This data was updated on a weekly basis and disseminated to the different stakeholders during the regular meetings in order to guide timely preventive and corrective measures. Surveillance findings related to vaccine-preventable diseases were shared with the national expanded immunization program on weekly basis and immediately if alert. Moreover, weekly reports/bulletins were generated and posted on the MOPH website. Of note, all ESU activities targeting Syrians in Lebanon were conducted in close collaboration with the different partners including UN agencies (WHO, UNHCR and UNICEF) and national or international NGOs through regular meetings, phone calls, and emails.

## 3. Results

During the study period, several communicable diseases were reported among Syrians, as presented in Table 1. The most commonly reported diseases were: viral hepatitis A, cutaneous leishmaniasis, mumps and measles (Table 1). No cases of poliomyelitis were reported.

**Figure 1:**
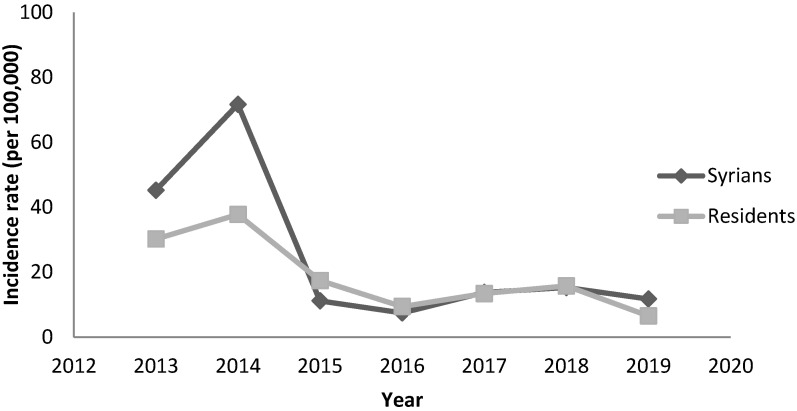
Annual incidence of hepatitis A by nationality, Lebanon, 2013–2019.

**Figure 2:**
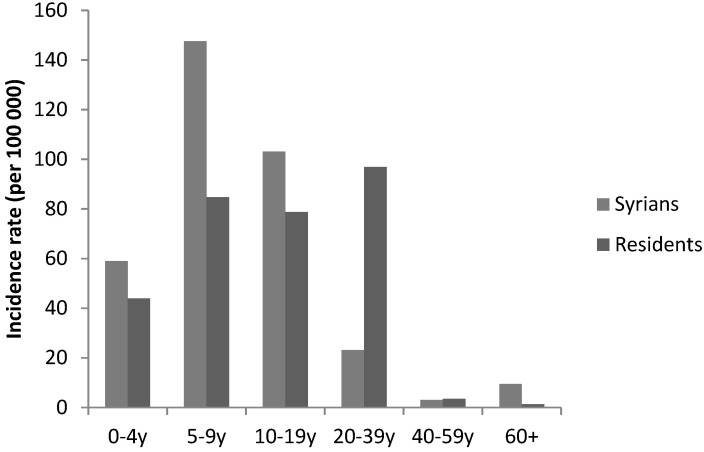
Annual incidence of hepatitis A by age group, Syrians, Lebanon, 2014.

Regarding hepatitis A, the annual incidence rate among Syrians reached 45 per 100,000 in 2013 and 72 per 100,000 in 2014. It decreased to less than 16 per 100,000 in the following years. In 2013–2014, a national outbreak was reported in Lebanon affecting residents and Syrians (Figure 1). During 2014, the highest incidence among Syrians was seen in the age groups 5–9 and 10–19 years (Figure 2) and among Syrians living in the Great Bekaa province.

**Figure 3:**
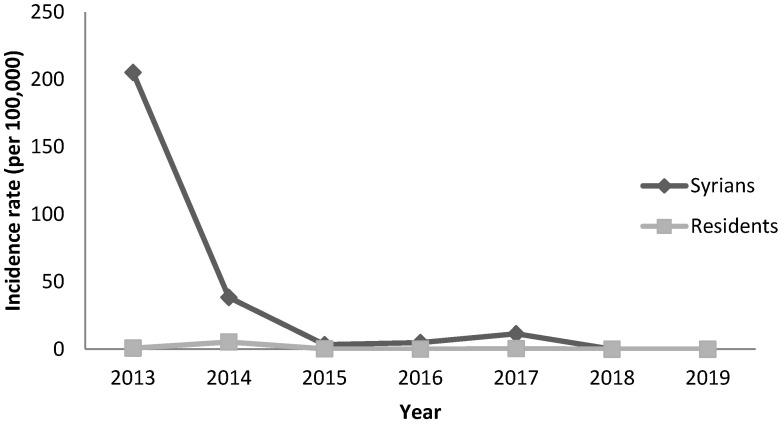
Annual incidence of leishmaniasis by nationality, Lebanon, 2013–2019.

**Figure 4:**
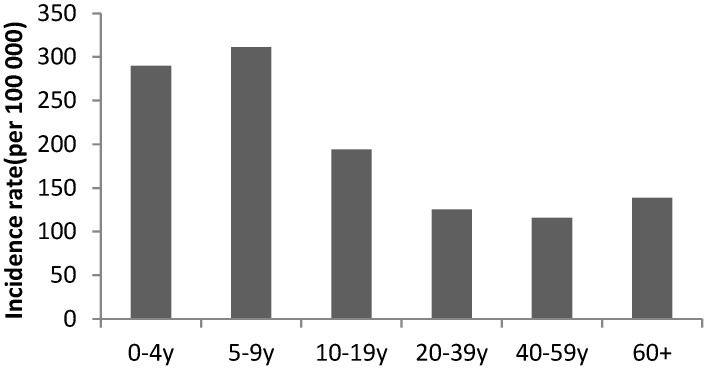
Annual incidence of leishmaniasis by age group, Syrians, Lebanon, 2013.

As for leishmaniasis, the incidence among Syrians reached 205 per 100,000 in 2013 then decreased to 39 per 100,000 in 2014 and to less than 12 per 100,000 in the following years. Syrians had highest rates when compared to residents (Figure 3). The highest incidence rate was among children under 10 years old (Figure 4). Cases were mainly reported from the Great Bekaa province and no transmission to local community was observed. 

**Figure 5:**
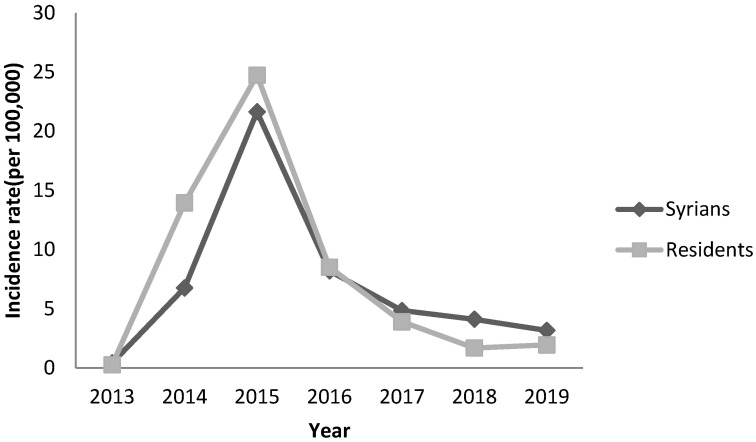
Annual incidence of mumps by nationality, Lebanon, 2013–2019.

**Figure 6:**
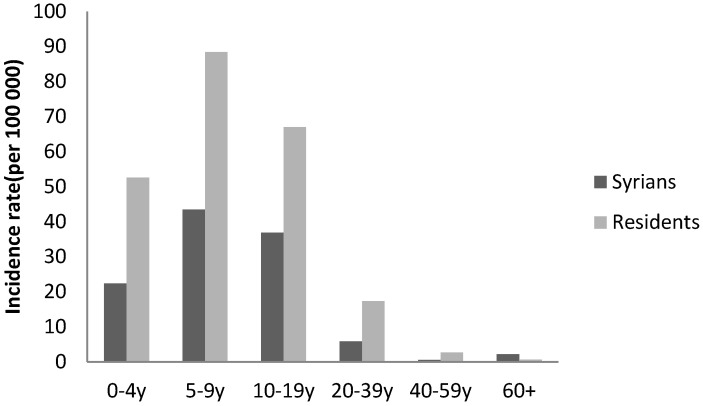
Annual incidence of mumps by age group and nationality, Lebanon, 2015.

The annual incidence of mumps among Syrians started at 0.41 per 100,000 in 2013, increased between 2014 and 2016 with a peak in 2015 reaching 21 per 100,000. In that year, a national outbreak was observed in Lebanon, affecting Syrians and the host population (25 per 100,000) equally (Figure 5). During 2015, the most affected age groups among Syrians were 5–9 years and 10–19 years (Figure 6). Cases were reported from the different provinces. 

**Figure 7:**
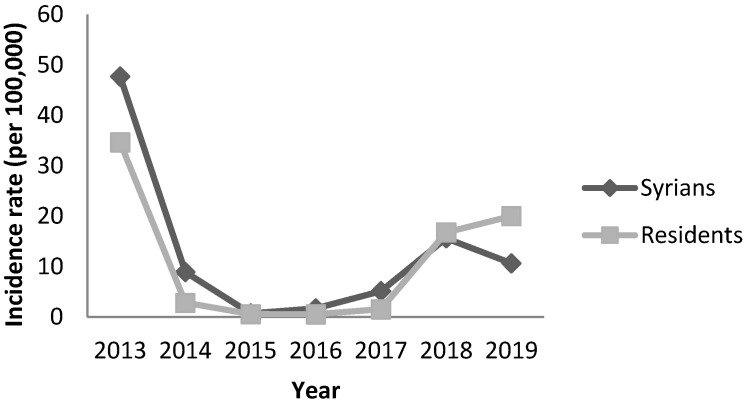
Annual incidence of measles by nationality, Lebanon, 2013–2019.

**Figure 8:**
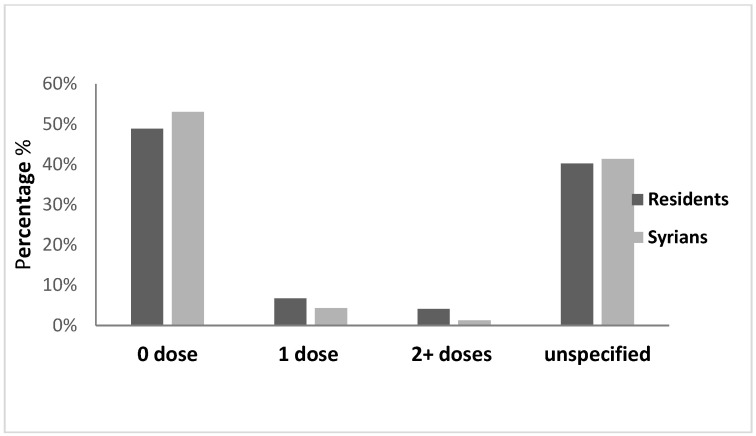
Distribution of measles cases by vaccination status and nationality, Lebanon, 2013.

**Figure 9:**
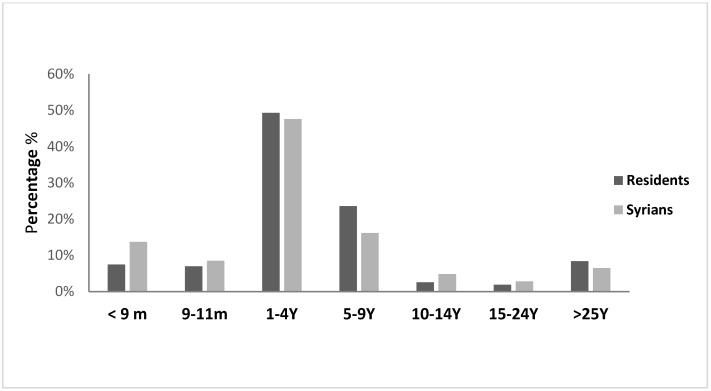
Distribution of measles cases by age group and nationality, Lebanon, 2018–2019. m: refers to months.

Outbreaks of measles were reported among Syrians in 2013 (48 per 100,000), 2018 (16 per 100,000) and 2019 (11 per 100,000) concomitant with the national measles outbreaks during these years (Figure 7). The most affected age groups were children aged less than 5 years old and 5–9 years. Forty nine percent of cases were not vaccinated for measles during both 2013 and 2018–2019 outbreaks while data on the vaccination status was not available for 40% and 36% of cases respectively. The distribution of cases by age groups and vaccination status were presented in Figure 8 and Figure 9. In 2013, the most affected provinces were the Great Bekaa and Mount Lebanon while in 2018–2019 outbreak, the most affected provinces were Great Bekaa and Great North. 

Regarding the AFP surveillance indicators, the non-polio AFP rate has dramatically increased after 2013 exceeding the target of 2 cases per 100,000 children <15 years of age and reaching a peak of 6 cases/100,000 in 2016. AFP cases detected during the study period were from different nationalities and all were discarded and classified as non-polio AFP cases. As for the AFP environmental surveillance, 168 specimens were collected during the study period from 4 sites. All were negative for wild poliomyelitis. 

## 4. Discussion

In this paper we describe surveillance activities and findings on communicable diseases reported in Lebanon after the Syrian displacement to Lebanon post 2011. Lebanon had a surveillance system in place which, with amendments, and with close collaboration with partners, was ready to monitor and detect outbreaks among all the population residing in Lebanon including the Syrian population. As shown in the findings, outbreaks of viral hepatitis A, leishmaniasis, mumps, and measles were reported.

The increase in viral hepatitis A, a viral disease affecting the liver and causing mild to severe illness and which is endemic in Syria [7], was reported among Syrians residing in Lebanon between 2013 and 2014. This could have been due to the poor living conditions which facilitate the rapid spread of diseases like crowded households, inadequate water supplies, lack of safe drinking water, and poor sanitation [3,7]. According to the WASH indicators of Vulnerability assessment of 2014, 33% of Syrian households did not have access to drinking water during that year and 22% did not have access to bathrooms at all. Out of those having access, 7% were sharing bathrooms and latrines with 15 persons or more [8]. However, a decrease in the incidence of hepatitis A was documented in the following years which could be attributed to the WASH activities implemented by UN agencies and NGOs [4]. For example, in another vulnerability assessment, conducted in 2018, showed that there had been improvements in provision of safe drinking water and access to sanitation facilities between 2015 and 2018. For instance, in 2018, 91% of refugee households reported having access to improved drinking water sources, 85% reported use of basic drinking water services. As for sanitation indicators, 87% of households reported having access to improved sanitation facilities and 68% use facilities which are not shared with other households [3]. Concerning similar reports in neighboring countries, a paper from Greece, revealed an increase of reported hepatitis A cases among refugees in 2016, where the majority were among Syrian refugees, and as in Lebanon, Greece worked on hygiene promotion and also vaccination [9]. 

Another important disease detected, which was considered scarce in Lebanon before 2013, was the cutaneous leishmaniasis, a vector born disease caused by a protozoa parasite transmitted through the bites of infected female phlebotomine sandflies. It causes skin lesions, mainly ulcers leading to life-long scars [10]. This disease is endemic in Syria with around 42,173 cutaneous leishmaniasis cases reported in 2010 and over 50,000 cases in 2015 [11]. In Lebanon, sporadic cases were reported between 2002 and 2012, with less than 10 cases reported annually among residents [12]. In 2013, however, ESU received reports of increased cases of cutaneous leishmaniasis throughout Lebanon. As a result, Leishmaniasis centers were set in selected public hospitals, dermatologists were trained, specimens were collected and sent to a reference histopathology laboratory, and treatment was provided. An increase in cases of leishmaniasis was also reported in neighboring countries that also received refugees such as in Jordan, Iraq and Turkey [13].

The increase of mumps incidence affected both Syrian and Lebanese populations. According to a case control study conducted by MOPH including Lebanese aged between 1.5 and 19 years old, the 2014–2015 mumps outbreak could be explained by the suboptimal uptake of the MMR vaccine and the accumulation of susceptible population. In this study, 94% of cases were not vaccinated compared to 51% of controls. It was also found in this study that overcrowding is a risk factor for mumps transmission during the outbreak and this could also explain the spread of the disease among Syrians in Lebanon [14]. Similarly, in another retrospective cohort study targeting schools in affected provinces, the MMR1 cohort vaccination coverage was 48% vs. 17% for MMR2 (unpublished). As a response to this outbreak, routine vaccination was enhanced for both host and displaced populations. Searching publications on similar outbreaks in neighboring countries brought back no results.

Further, the measles outbreak witnessed in 2013 and 2014 in Lebanon also appeared to have hit all populations in Lebanon. Measles is a highly contagious, serious disease causing an estimated 2.6 million deaths each year [15]. Although Lebanon adopted the WHO strategic plan to achieve measles elimination, measles outbreaks occur every few years: between 1997–1998 and then yearly from 2003 to 2007, affecting the different Lebanese provinces. During this study period, the numbers of measles cases were slightly higher among the Lebanese population, yet, Syrians were significantly affected. Lack of vaccination among children appeared to have been the major cause of the outbreak. Hence, as a response, accelerated vaccination activities were conducted by the Expanded Immunization Program (EPI) in close collaboration with UNICEF and WHO in addition to a vaccination campaign conducted in 2019–2020. In Syria during the same time in June 2013, up to 7000 measles cases were seen in districts in northern Syria, according to a report published by MSF [16] and 1617 suspected cases were reported in 2015 [11]. Clusters of measles in neighboring countries have been reported. For example, Turkey and Jordan reported 625 and 205 cases among Syrian refugees in 2013 [13].

Finally, no cases of poliomyelitis have been detected in Lebanon during the study period although Lebanon was considered at high risk of having polio cases due to the outbreak of polio in Syria. Poliomyelitis which is a highly infectious disease mainly affecting children less than 5 years old is transmitted from person to person through the oral-fecal route leading to paralysis [17]. Syria was polio free from 1999 till 2013–2014 when a wild poliovirus outbreak was declared in the country leading to 36 paralyzed cases [18]. In Lebanon, the last two indigenous polio cases were reported in 1994 and Lebanon was declared polio free in 2002 and has been ever since. Remaining polio free was mainly due to the enhanced acute flaccid paralysis surveillance and the successive national vaccination campaigns targeting both host and Syrian populations.

### 4.1. Strengths

Flexibility is one of the important attributes of surveillance systems; it reflects systems that can adjust to circumstances with the available resources [19,20]. According to a health system resilience study, the Lebanese surveillance system was able to adjust and function properly within the context of the Syrian displacement in Lebanon [20]. Also based on our findings, outbreaks were detected despite Lebanon having hosted the highest number of refugees per capita at that time. Further, in terms of data quality, a WHO assessment mission for the national surveillance system and laboratory capacity in Lebanon was conducted in 2016 and revealed surveillance indicators of completeness and timeliness from health facilities ranged between 93% and 83%, which is adequate for a proper surveillance system [19]. Additionally, technical and financial assistance helped the surveillance program maintains its activities and response programs such as vaccination and hygiene promotion were possible. 

### 4.2. Challenges and Limitations

In terms of challenges and limitations, it is important to note a few that might have affected this study. A practical challenge before the year 2017, was that Lebanon relied on the fax for receiving reports and this was found to delay the process of reporting, as was also reported in Italy [21]. However, this was overcome by the shift to electronic reporting using the DHIS2 system which improved reporting significantly and was also adapted by MMUs in the field reporting on refugee health outcomes. 

Moreover, the real size of Syrian refugee population compared to the one registered was a challenge for determining the denominator for incidence estimations. Further, this study does not report all communicable diseases present in Lebanon for example Tuberculosis and HIV/AIDs are surveyed and managed by vertical programs at the MOPH and hence their numbers are not reflected here. Another limitation is in relation to the Lebanese healthcare system which is a competitive market-driven healthcare system, dominated by the private sector that constitutes 90% of all hospital beds in Lebanon [5,6]. Though the MOPH does cover uninsured residents in Lebanon, still the out-of-pocket expenditure is unfortunately high (53%), affecting health outcomes of all residents in Lebanon especially refugees after coverage by UNHCR decreased throughout the years [7,8]. This could have affected the health seeking behavior of residents and refugees affecting indirectly the completeness of the reported data. Finally, in 2020 until this date, due to the COVID-19 pandemic, healthcare facilities and MOPH teams were overwhelmed, affecting surveillance of communicable diseases for both Syrians and host communities and hence our numbers do not include this time period. 

## 5. Conclusions

This study aimed to provide the public with an understanding of how the Lebanese surveillance program functioned and to share data of the program after and during the Syrian displacement to Lebanon. Despite some limitations and challenges, communicable diseases have been monitored among Syrians in Lebanon, and outbreaks have been detected and investigated. The existence of an already implemented surveillance system has helped to monitor the health status of Syrians in Lebanon, early detect communicable diseases among this population and guide needed preventive and control measures. This highlights the importance of having a flexible surveillance system that can be adapted to emergency situations and the importance of sharing results with involved partners. It would be beneficial if all countries facing the same displacement report their activities for experience sharing and learning lessons.

## Figures and Tables

**Table 1:** Reported communicable diseases among Syrians, Lebanon, 2013–2019.

Disease	Year							Total
	2013	2014	2015	2016	2017	2018	2019	
Viral hepatitis A	220	859	182	78	139	152	111	1741
Leishmaniasis	998	461	56	52	116	0	0	1683
Measles	232	107	12	18	52	156	101	678
Mumps	2	81	354	86	49	41	30	643
Brucellosis	12	35	85	165	149	26	32	504
Viral hepatitis B	8	31	53	48	52	28	36	256
Malaria	1	5	3	0	75	82	80	246
Meningitis	24	34	61	63	0	0	0	182
Food poisoning	11	30	16	28	74	14	0	173
Typhoid fever	21	26	70	11	19	4	6	157
Pertussis	9	25	8	18	22	26	23	131
Acute flaccid paralysis	7	12	10	17	19	33	18	116
Rubella	1	12	2	6	6	4	9	40
Viral hepatitis C	4	5	6	8	0	0	0	23
Rabies	1	0	0	0	1	1	0	3
Leprosy	0	1	0	1	0	0	0	2
Tetanus	0	0	0	0	0	1	0	1
Gonorrhea	0	0	1	0	0	0	0	1
Syphilis	0	0	1	0	0	0	0	1
Poliomyelitis	0	0	0	0	0	0	0	0

## Data Availability

Publicly available datasets were analyzed in this study. This data can be found here: https://moph.gov.lb/en/Pages/2/193/esu (accessed on 15 June 2022).
